# Screening Performance Characteristics of Ultrasonography in Confirmation of Endotracheal Intubation; a Systematic Review and Meta-analysis 

**DOI:** 10.22037/aaem.v9i1.1360

**Published:** 2021-10-26

**Authors:** Mehrdad Farrokhi, Bardia Yarmohammadi, Amir Mangouri, Yasaman Hekmatnia, Yaser Bahramvand, Moein Kiani, Elham Nasrollahi, Milad Nazari-Sabet, Niusha Manoochehri-Arash, Maria Khurshid, Shima Mosalanejad, Vida Hajizadeh, Reza Amani-Beni, Masoumeh Moallem, Maryam Farahmandsadr

**Affiliations:** 1 Eris Research Institute, Tehran, Iran.; 2 School of Medicine, Shahid Beheshti University of Medical Sciences, Tehran, Iran.; 3 Department of Vascular and Endovascular Surgery, Sina Hospital, Tehran University of Medical Sciences, Tehran, Iran.; 4 Islamic Azad University, Sari Branch, School of Medicine, Sari, Iran.; 5 School of Medicine, Shiraz University of Medical Sciences, Shiraz, Iran.; 6 School of Medicine, Zanjan University of Medical Sciences, Zanjan, Iran.; 7 Department of General Surgery, School of Medicine, Isfahan University of Medical Sciences, Isfahan, Iran.; 8 Endocrine Research Center, Research Institute for Endocrine Sciences, Shahid Beheshti University of Medical Sciences, Tehran, Iran.; 9 Department of Internal Medicine, Berkshire Medical Center, Pittsfield, Massachusetts, USA.; 10 Department of Internal Medicine, Faculty of Medicine, Tehran Medical Sciences, Islamic Azad University, Tehran, Iran.; 11 School of Dentistry, Shiraz University of Medical Sciences, Shiraz, Iran.; 12 School of Medicine, Isfahan University of Medical Sciences, Isfahan, Iran.; 13 Department of Emergency Medicine, School of Medicine, Tehran Medical Sciences Branch, Islamic Azad University, Tehran, Iran.; 14 School of Medicine, Jahrom University of Medical Sciences, Jahrom, Iran.

**Keywords:** Airway management, intubation, meta-analysis, sensitivity and specificity, ultrasonography

## Abstract

**Introduction:**

Recent studies have suggested that point-of-care ultrasonography can be used for confirming the placement of endotracheal tube. This systematic review and meta-analysis aimed to investigate the sensitivity and specificity of ultrasonography for confirming endotracheal tube placement.

**Methods:**

In this meta-analysis, systematic search of the previous published papers investigating the diagnostic accuracy of ultrasonography for confirmation of endotracheal tube placement was performed. Seven electronic databases, including PubMed, Scopus, Google Scholar, EBSCO, EMBASE, Web of Science, and Cochrane Database were searched up to July 2021, for all relevant articles published in English on this topic. Meta-DiSc version 1.4 software was used for statistical analysis.

**Results:**

The estimated pooled sensitivity and specificity of ultrasonography for confirmation of endotracheal tube location were 0.98 (95% CI: 0.98–0.99) and 0.94 (95% CI 0.91–0.96), respectively. The pooled positive likelihood ratio and negative likelihood ratio were 5.94 (95% CI 4.41–7.98) and 0.03 (95% CI: 0.02-0.04), respectively. The diagnostic odds ratio of ultrasonography was 281.47 and the area under hierarchical summary receiver operating characteristic (HSROC) revealed an appropriate accuracy of 0.98.

**Conclusion:**

Ultrasonography has high diagnostic accuracy and can be used as a promising tool for confirmation of endotracheal tube placement, especially in critically ill patients or when capnography is not available, or its result is equivocal.

## 1. Introduction

Securing a definitive airway in critically ill patients is a necessary procedure performed in intensive care unit (ICU), out of hospital, and in the emergency department (ED) settings. Direct assessment of the endotracheal tube passage through the cords is commonly performed via primary localization, followed by a confirming method (1, 2). However, direct visualization of endotracheal tube passing through the cord may be misleading during difficult intubations, which may lead to esophageal intubation in emergency cases. Unrecognized esophageal intubations are associated with catastrophic consequences such as neurological complications or death. Therefore, different techniques are often used to confirm the appropriate placement of endotracheal tube, but not all of them are all of them are not reliable enough to confirm the tracheal intubation (3, 4). It has been suggested that both clinical evaluations and confirmatory methods including auscultation, chest expansion following ventilation, bronchoscopy, chest X-ray, capnography, and end-tidal carbon dioxide (ETCO2) assessment should be used to confirm the location of endotracheal tube. In this regard, ETCO2 has not been suggested for patients with cardiac arrest or embolism. Similarly, capnography has some limitations, including low reliability in patients with embolism or cardiac arrest, or recent bag-valve-mask use (5-7). Due to the above-mentioned limitations, combined with growing application of ultrasound by emergency medicine (EM) physicians and ease of use of point-of-care ultrasonography, many studies have been performed to assess the reliability of ultrasonography for approving the placement of endotracheal tube. However, most of these investigations had small sample sizes with different gold standards, resulting in conflicting findings. Accumulating lines of evidence have recently suggested that point-of-care ultrasonography can be used as an adjunct for confirming the placement of endotracheal tube, especially in critical situations such as cardiac arrest or when other confirmation methods are not available (8-10). However, before approval of ultrasonography as a promising technique for confirmation of endotracheal tube placement, it is necessary to pool the results of previously published studies. Therefore, in this systematic review and meta-analysis, we aimed to assess the screening performance characteristics of ultrasonography in confirmation of endotracheal tube placement. 

## 2. Methods

**2.1. Data Sources and Searches**

We performed a systematic search of the previous published papers investigating the diagnostic accuracy of ultrasonography for confirmation of endotracheal tube placement. We searched PubMed, Scopus, Google Scholar, EBSCO, EMBASE, Web of Science, and Cochrane Databases from inception to July 2021. The systematic search was carried out using medical subject heading (MeSH) terms for ''ultrasonography'' and ''intubation''. In this regard, we used ''sono'', ''sonography'', ''ultrasonography'', ''ultrasound'', ''endotracheal intubation'', ''esophageal intubation'', and ''intubation''. Our search had no restrictions with respect to location of study or publication date. Furthermore, in this meta-analysis, we only assessed human studies. 

2.2. Selection Criteria

In this meta-analysis we included studies investigating the diagnostic accuracy of bedside ultrasound to confirm endotracheal tube placement following emergency or elective intubation in adult subjects. The included studies were required to compare the findings of ultrasonography with a gold standard technique, such as fiberoptic bronchoscopy or capnography, for confirmation of endotracheal tube placement. Retrospective design studies, case reports, case series, and reviews were excluded and clinical trials, case-control or cohort design studies were included in this study. Investigations performed using mannequins, cadavers, or pediatric patients were excluded from the study. Two independent reviewers (M.F and B.Y) assessed the studies according to the above-mentioned criteria and any discrepancy between them was resolved by a third reviewer (M.K). In order to avoid possible duplicates, we searched the first author’s name, as well as the place and the period of the subjects’ enrolment. In the case of different versions of the same study, only the most recent was considered.

**2.3. Data Extraction**

Data were extracted by two reviewers and included characteristics of the studies (the first author, publication date, sample size, male percentage, mean age of participants, and location of intubation), ultrasonic technique, transducer type, percentage of esophageal intubation, gold standard for confirmation of endotracheal tube placement, and diagnostic accuracy parameters of ultrasonography (number of true positive, true negative, false positive, and false negative). The process of data extraction was performed by two investigators independently and finally, inconsistencies regarding included studies were resolved by a third reviewer. 

**2.4. Data Synthesis**

Meta-DiSc version 1.4 software and Comprehensive Meta-Analysis software version 3 were used for statistical analysis. The heterogeneity among the included studies was investigated using Q-statistic and I^2^ index. If the value of I^2^ was higher than 50% or P-value was less than 0.10, the random model was used to estimate the sensitivity and specificity of ultrasonography for confirmation of endotracheal tube placement. Alternatively, if the value of I^2^ was less than 50% and P-value was higher than 0.10, the sensitivity and specificity of ultrasonography were calculated using fixed model. Egger’s test and funnel plot were used to evaluate publication bias.

## 3. Results

**3.1. Search Results**

**Figure 1:**
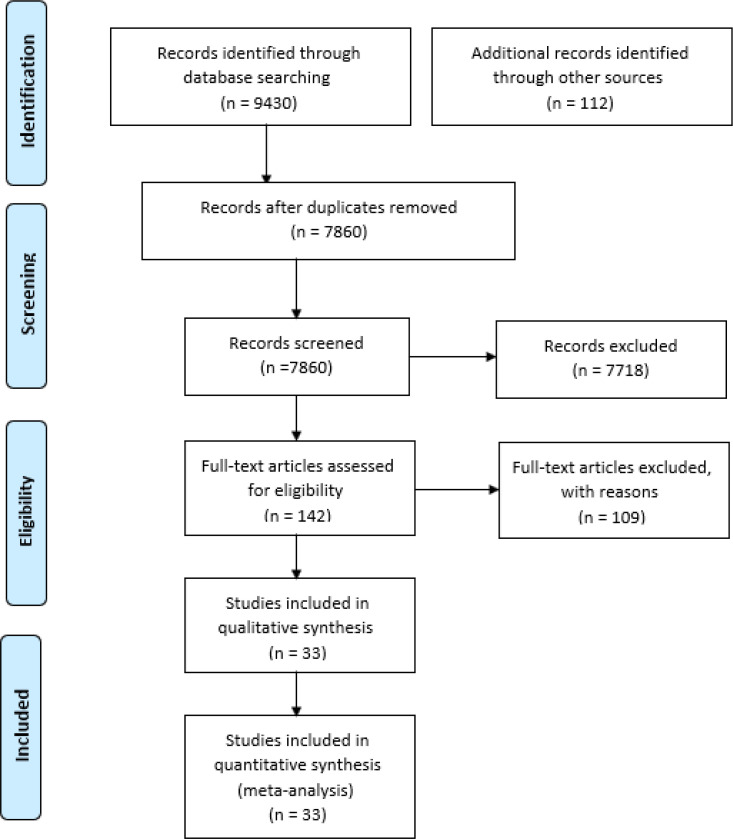
PRISMA flowchart of the literature search and selection of studies that reported accuracy of ultrasonography for confirmation of endotracheal placement

Figure 1 summarizes the flow of studies in this review according to Preferred Reporting Items for Systematic Reviews and Meta-Analyses (PRISMA) recommendations. A total of 9542 studies were identified in our preliminary search. After removal of 1682 duplicates, abstracts of the remaining 7860 studies were assessed by two independent reviewers (M.F and B.Y). The full-text of 142 articles were evaluated for eligibility and 107 article were excluded according to the exclusion criteria. Finally, 33 articles evaluating 2840 patients were included in our meta-analysis.

**3.2. Characteristics of Included Studies**

**Figure 2:**
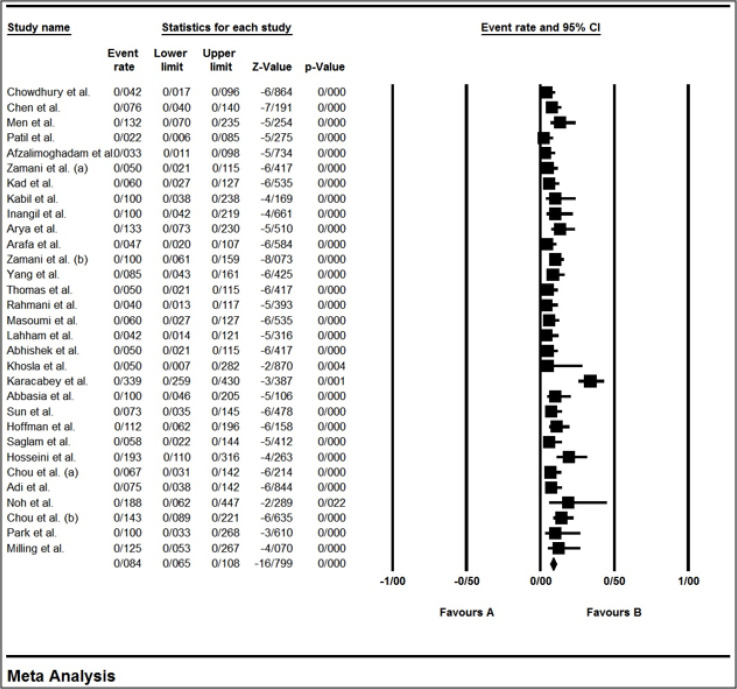
Forest plot of prevalence of esophageal intubation

The characteristics of the 33 included studies are summarized in table 1. Studies were performed between 2007 and 2020, with the sample sizes ranging from 19–150 subjects. Most of the included studies were performed in Iran (seven studies). Twenty-nine studies were prospective observational studies and 4 were controlled trials. Five studies were conducted in ICUs, 10 were performed in operating rooms, and 18 were carried out in EDs. The prevalence of esophageal intubation was estimated to be 8.4% (95% CI: 6.5-10.8; Figure 2).

**3.3. Publication Bias and Quality Assessment**

**Figure 3:**
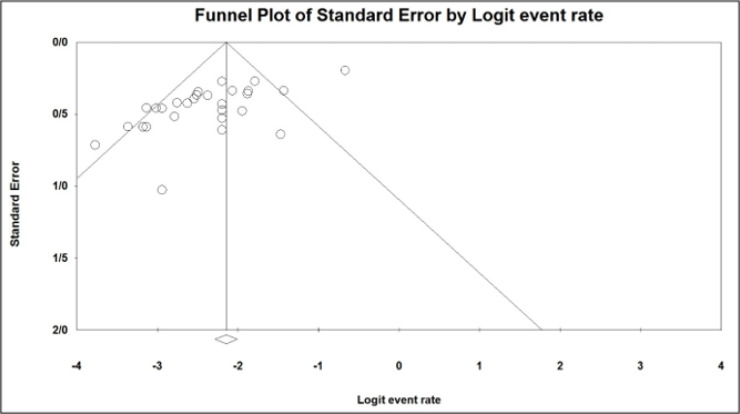
Publication bias of the included studies for analysis of the rate of esophageal intubation confirmed using ultrasonography

Assessment of publication bias based on Egger's test showed that there was a statistically significant publication bias (P<0.01). Moreover, the funnel plot of included studies revealed significant asymmetry (Figure 3). Quality assessment of the included studies was performed using QUADAS-2 tool (Table 2).

**3.4. Diagnostic Accuracy Indices**

**Figure 4:**
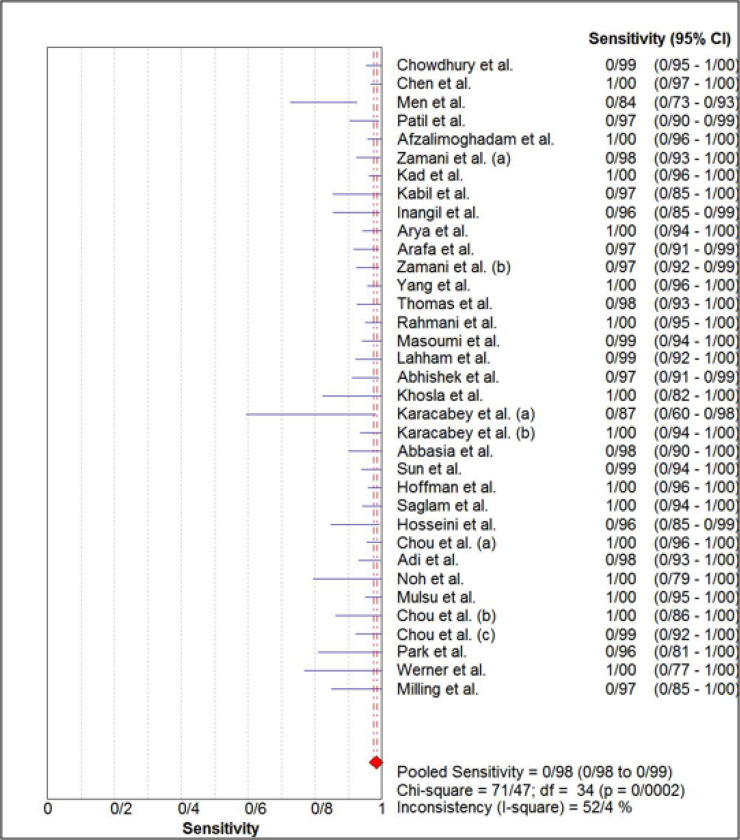
Forest plot of the overall sensitivity of ultrasonography for confirmation of endotracheal tube placement

**Figure 5:**
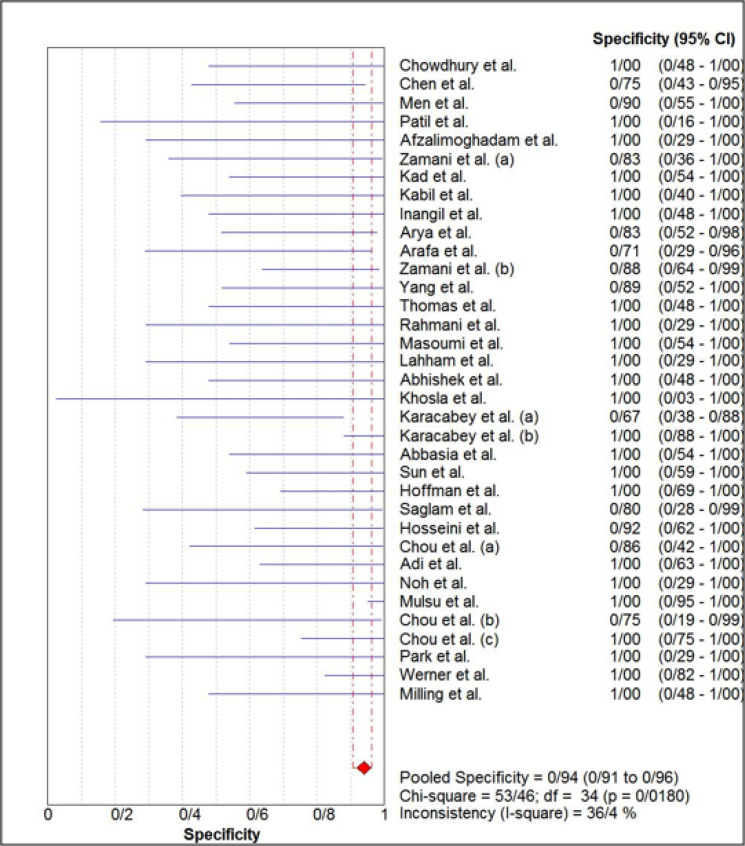
Forest plot of the overall specificity of ultrasonography for confirmation of endotracheal tube placement

**Figure 6:**
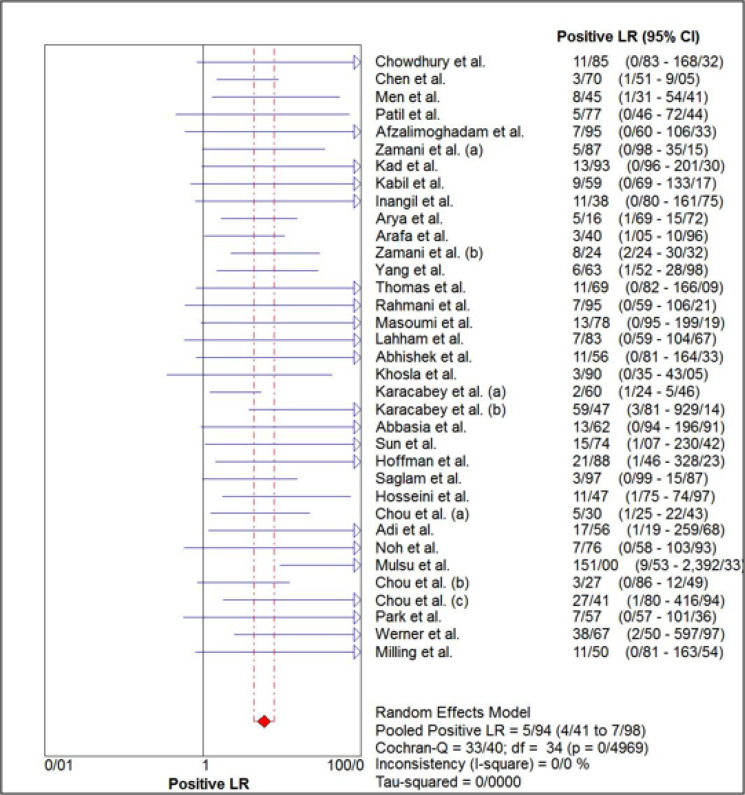
Forest plot of the overall positive likelihood ratio of ultrasonography for confirmation of endotracheal tube placement

**Figure 7:**
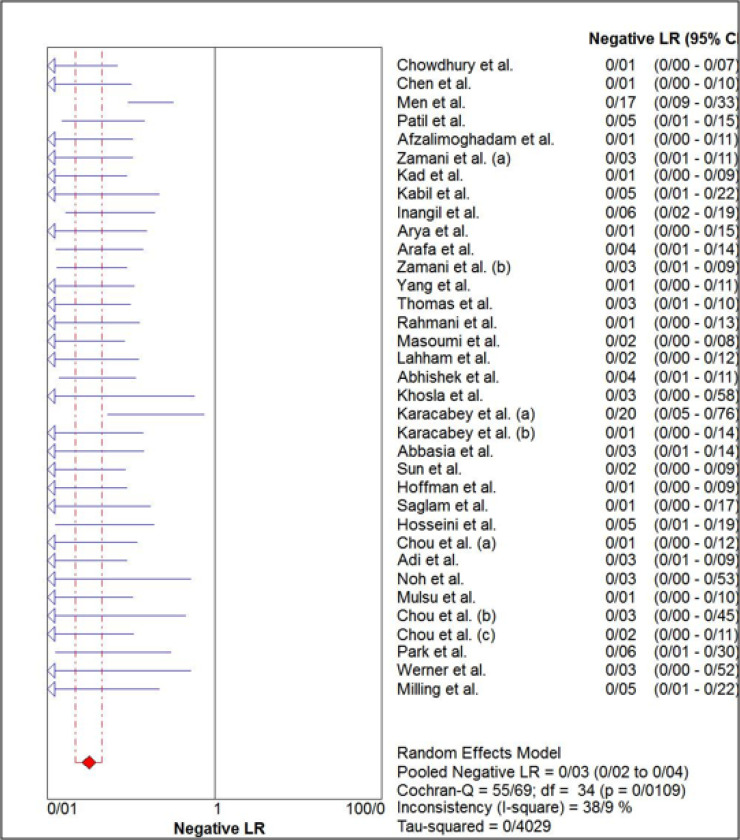
Forest plot of the overall negative likelihood ratio of ultrasonography for confirmation of endotracheal tube placement

**Figure 8:**
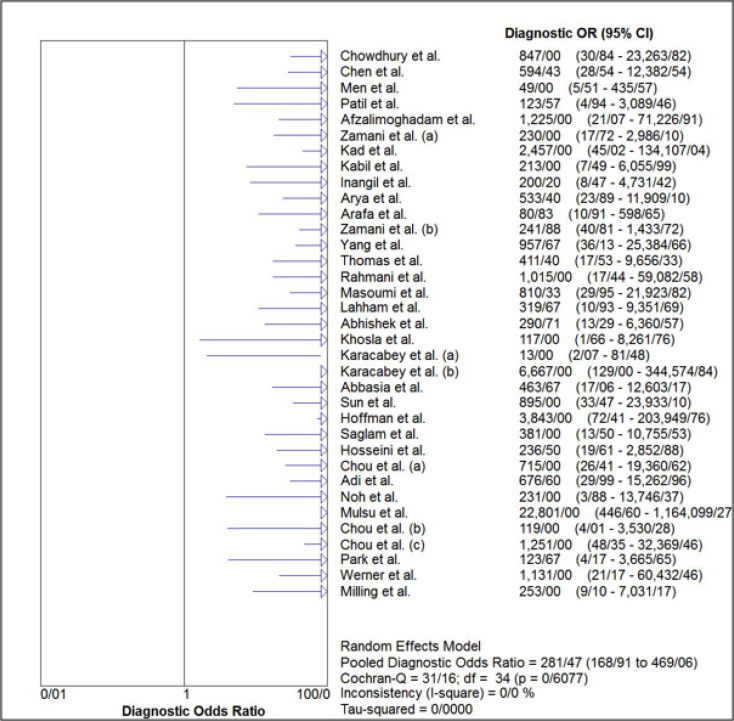
Forest plot of the overall diagnostic odds ratio (OR) of ultrasonography for confirmation of endotracheal tube placement

**Figure 9:**
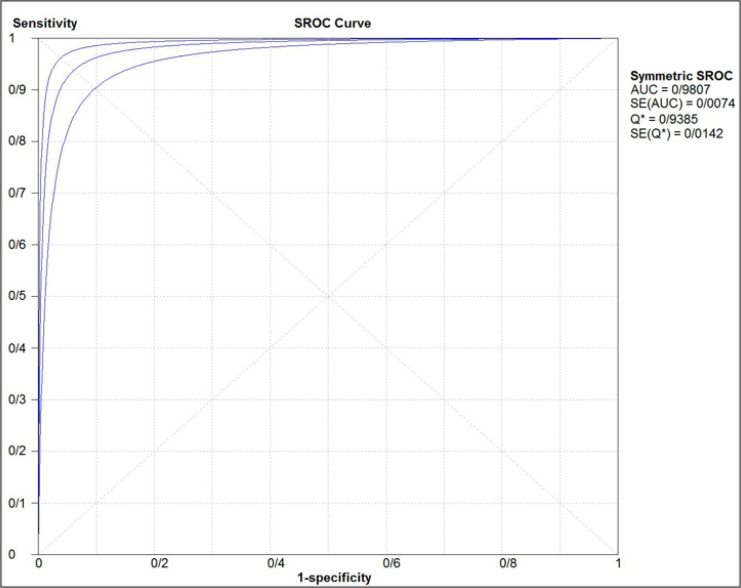
Hierarchical summary receiver-operating characteristic curve (HSROC) indicating accuracy of ultrasonography for confirmation of endotracheal tube placement

The estimated pooled sensitivity and specificity of ultrasonography for confirmation of endotracheal tube location were 0.98 (95% CI: 0.98–0.99) and 0.94 (95% CI 0.91–0.96), respectively (Figure 4 and Figure 5). The pooled positive likelihood ratio and negative likelihood ratio were 5.94 (95% CI 4.41–7.98) and 0.03 (95% CI: 0.02-0.04), respectively (Figure 6 and Figure 7). Furthermore, the diagnostic odds ratio of ultrasonography was 281.47 (95% CI: 168.91–469.06) (Figure 8). The area under hierarchical summary receiver operating characteristic curve (HSROC) revealed an appropriate accuracy of 0.98 (Figure 9). Subgroup analysis based on transducer type and location of intubation (ICU or ED, or operating room) showed acceptable sensitivity and specificity. 

## 4. Discussion

The results showed that the estimated pooled sensitivity and specificity of ultrasonography for confirmation of endotracheal tube location were 0.98 and 0.94, respectively. The diagnostic odds ratio of ultrasonography was 281.47 and the area under HSROC revealed an appropriate accuracy of 0.98.

Our findings confirm the efficacy of ultrasonography as an adjunct for assessment of endotracheal tube location during intubation. It should be noted that these results are important since capnography has been considered to have low accuracy, especially in subjects with critical conditions. Similarly, ultrasonography has been approved by advanced cardiac life support guidelines as an adjunct for capnography to confirm endotracheal tube placement (11). Furthermore, most confirmatory techniques need some ventilation, which is associated with higher rates of aspiration and gastric distention in cases with wrong location of endotracheal tube (5, 12). On the other hand, ultrasonography does not increase risk of aspiration or gastric distention and has some advantages including being available in different locations, noninvasive, and rapid for confirmation of endotracheal tube placement.

In a similar study, Adhikari et al. (13) have performed a systematic search in different databases to identify studies evaluating efficacy of ultrasonography for confirmation of endotracheal tube placement. Their systematic search yielded 5 eligible studies. In this study, the authors estimated 91% sensitivity (95% CI, 74% to 97%) and 97% specificity (95% CI, 89%to 99%) for ultrasonography, which are lower than those found in our study. 

In another study, a systematic search was carried out in EMBASETM, MEDLINE, LILACS, The Cochrane Library, KoreaMed, OpenGrey, and the World Health Organization International Clinical Trials Registry from their inception to 2014, which yielded 11 studies with 969 patients (14). They reported pooled sensitivity and specificity of ultrasonography in confirming the placement of the tube as 0.98 and 0.98, respectively. Although their estimated pooled sensitivity was similar to that found in our study, their pooled specificity was higher than ours. These differences can partially be explained by the difference in the number of included studies, sample sizes of patients, and also causes of patient hospitalization.

Although our findings confirmed the efficacy of ultrasonography as a promising adjunct for confirmation of endotracheal tube placement, it should be noted that there are some significant limitations for ultrasonography. First, the efficacy of ultrasonography is dependent on the operator and ultrasonography by different operators may result in different ultrasonographic image qualities and decisions. Therefore, ultrasonography operators must obtain necessary skills before performing ultrasonography for confirmation of endotracheal tube location. Furthermore, ultrasonography cannot be performed easily in situations where there is only one operator, because that operator may be the technician performing endotracheal intubation. Therefore, in these cases, the static techniques is superior to dynamic technique. Moreover, the placement of ultrasonographic transducer on the trachea when a tube is entering the trachea may be associated with a more difficult intubation as it might deviate its path. In this regard, it has been suggested that the pressure of transducer on trachea should be reduced by ultrasonography operator to prevent deviation of endotracheal tube during intubation. If ultrasonography increases the risk of difficult intubation, the procedure should be performed using static technique. From another point of view, ultrasonography may be difficult for some intubations including cases with different airway anatomy, neck edema, cervical collar, subcutaneous emphysema, and neck masses. Unlike ultrasonography, capnography requires four to five ventilations to confirm the location of endotracheal tube placement, so ultrasonography is faster than capnography. However, the efficacy of capnography is not dependent on the experience of operator and training does not affect the accuracy of this method (15). 

It is now well established that ultrasonography has several strong points for confirmation of endotracheal tube location. Deviation of endotracheal tube into the esophagus can be easily identified before initiation of ventilation because ultrasonography is carried out in real time during intubation. Since ultrasonography has an appropriate specificity for identification of esophageal intubation, this method can be used in cases with indefinite result of capnography to reduce the total number of intubation attempts. Furthermore, ultrasonography does not interfere with chest compression and intubation can be performed during cardiopulmonary resuscitation. However, further studies with larger sample sizes using appropriate gold standards are required to establish ultrasonography as a promising diagnostic test for assessment of endotracheal tube location.

**Table 1:** Characteristic of studies included in the meta-analysis

**Gold** **Standard**	**Esophageal ** **Intubation (%) **	**Transducer** **Type**	**Ultrasonic ** ** Technique **	**Male ** **(%)**	**Mean ** **Age**	**Location**	**Sample ** **Size**	**Year**	**Author**
CAP+A	4.1	Linear	Dynamic	28	39.02	OR	120	2020	Chowdhury et al. (3)
DV+FB	10.2	NR	Dynamic	60.2	71.5	ICU	118	2020	Chen et al. (1)
A+FB	17.6	Curvilinear	Static	54	60.4	OR	68	2019	Men et al. (16)
CAP	2	Linear	Dynamic	NR	NR	ICU	91	2019	Patil et al. (17)
CAP	3.3	Linear	Dynamic	58.9	59.2	ED	90	2019	Afzalimoghadam et al. (18)
CAP	6	Linear	Dynamic	73	57.5	ED	100	2018	Zamani et al. (19)
A	2	Linear	Static	NR	NR	OR	100	2018	Kad et al. (20)
FB	10	Curvilinear	Dynamic	65	55.7	ICU	40	2018	Kabil et al. (21)
CAP	6	Linear	Dynamic	56	42.9	OR	50	2018	Inangil et al. (22)
CAP	16	Linear	Dynamic	55.3	63.4	ICU	75	2018	Arya et al. (23)
CAP	7.5	Linear	Dynamic	63.6	41.4	OR	107	2018	Arafa et al. (24)
O+AS+DV+A	11.3	Linear	Static	56	58.5	ED	150	2017	Zamani et al. (25)
CAP+A	9.7	Linear	Static	46	53.5	OR	93	2017	Yang et al. (26)
CAP	5	Linear	Static	59	50.8	ED	100	2017	Thomas et al. (27)
DV	0	Linear	Dynamic	62.7	61.1	ED	75	2017	Rahmani et al. (28)
CAP	6	Curvilinear	Static	65	64.5	ED	100	2017	Masoumi et al. (8)
CAP	4.2	Linear	Dynamic	56.9	57.7	ED	72	2017	Lahham et al. (9)
CAP	5	Linear	Static	NR	38.9	OR	100	2017	Abhishek et al. (5)
CAP+A	0	Linear	Static	100	70.5	ICU	20	2016	Khosla et al. (29)
CAP	38.2	Linear	Dynamic	NR	67.2	ED	85	2016	Karacabey et al. (a) (10)
CAP	NR	Linear	Dynamic	NR	NR	ED	30	2016	Karacabey et al. (b) (10)
DV+A+CAP	11.7	Linear	Dynamic/Static	61.5	50	ED	120	2015	Abbasia et al. (30)
CAP+A	7.3	Curvilinear	Dynamic	67.6	68.8	ED	96	2014	Sun et al. (31)
DV+CAP	10	Linear	Dynamic/Static	NR	58	ED	101	2014	Hoffman et al. (32)
CAP	7.2	Linear	Static	NR	NR	ED	69	2013	Saglam et al. (33)
DV+A+O	21	Curvilinear	Static	60	59	ED	57	2013	Hosseini et al. (34)
CAP+A	7.6	Curvilinear	Dynamic	69	69.9	ED	89	2013	Chou et al. (a) (35)
CAP	5.6	Linear	Static	NR	NR	ED	107	2013	Adi et al. (36)
CAP	15.7	Linear	Dynamic	NR	NR	ED	19	2012	Noh et al. (37)
CAP+A	50	Linear	Dynamic	50.7	40.5	OR	150	2011	Mulsu et al. (38)
CAP	15.7	Curvilinear	Static	54.5	67.6	ED	83	2011	Chou et al. (b) (39)
CAP	10.3	Curvilinear	Static	NR	NR	ED	29	2011	Chou et al. (c) (39)
CAP+A	10	Linear	Dynamic	56.7	59.6	ED	30	2009	Park et al. (40)
DV+CAP	57.6	Linear	Dynamic	21.2	38.9	OR	66	2007	Werner et al. (41)
CAP+A	12.5	Curvilinear	Dynamic	17.5	52.5	OR	40	2007	Milling et al. (42)

OR: operation room; ICU: intensive care unit; ED: emergency department; NR: not reported; CAP: capnography; A: auscultation; DV: direct visualization; FB: fiberoptic bronchoscopy; O: ; AS: .

**Table 2:** Quality assessment of the included studies using QUADAS-2 tool

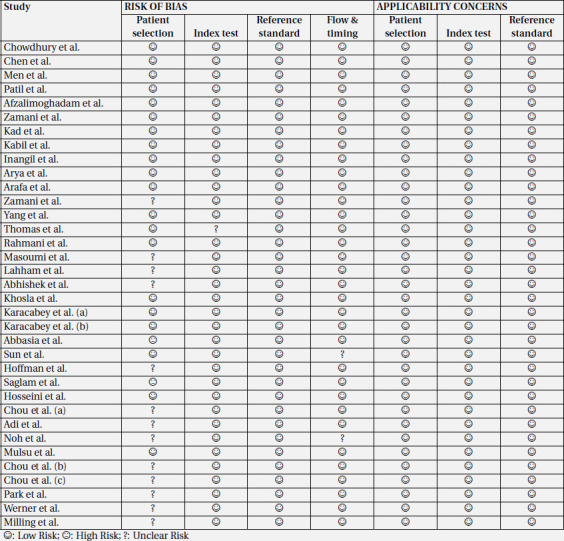

## 5. Limitations

Different methods of confirmation were used as gold standard to indicate sensitivity and specificity of sonography for confirmation of endotracheal tube placement.

## 6. Conclusion

The results showed that ultrasonography has high diagnostic accuracy and can be used as a promising tool for confirmation of endotracheal tube placement, especially in critically ill patients or when capnography is not available, or its result is equivocal.

## 7. Declarations

### 7.1. Acknowledgement

The authors thank all those who contributed to this study.

### 7.2. Author Contribution

All authors contributed to study design, data collection, writing draft of study.

### 7.3. Funding/Support

None.

### 7.4. Conflict of interest

None.

### 7.5. Data Availability

Not applicable. 
